# A Prospective Analysis of Viscoelastic Assays, Platelet Aggregometry, and Standard Laboratory Tests in Predicting Perioperative Blood Loss in Cardiac Surgery

**DOI:** 10.1177/10760296261432450

**Published:** 2026-03-05

**Authors:** Yerlan Orazymbetov, Serik Aitaliyev, Povilas Jakuška, Audronė Veikutienė, Tadas Lenkutis, Rassul Zhumagaliyev, Vilius Skipskis, Yerik Aitaliyev, Aušra Saudargienė, Rimantas Benetis

**Affiliations:** 1 Department of Cardiac, Thoracic and Vascular Surgery, Hospital of Lithuanian University of Health Sciences Kauno Klinikos, 230647 Lithuanian University of Health Sciences, Kaunas, Lithuania; 2 Department of Cardiac Surgery, National Scientific Medical Centre, Astana, Kazakhstan; 3 230647 Institute of Cardiology, 230647 Lithuanian University of Health Sciences, Kaunas, Lithuania; 4 Department of Anaesthesiology, Medical Academy, 230647 Lithuanian University of Health Sciences, Kaunas, Lithuania; 5 186040 West Kazakhstan Medical University, Aktobe, 030019, Kazakhstan; 6 Neuroscience Institute, 230647 Lithuanian University of Health Sciences, Kaunas, Lithuania, Kaunas, Lithuania

**Keywords:** cardiac surgery, Hb/kg index, thromboelastometry, platelet aggregation, fibrinogen

## Abstract

**Background:**

Postoperative bleeding following cardiopulmonary bypass (CPB) remains a significant challenge. Although viscoelastic testing is increasingly used, the relative contributions of fibrinogen, platelet count and clot firmness to blood loss remain debated. We evaluated the diagnostic accuracy of thromboelastometry (ROTEM) compared with platelet aggregometry (PA) and standard tests, using the Hb/kg index to quantify blood loss.

**Methods:**

In this prospective observational study conducted at the University Hospital (Kaunas, Lithuania) we enrolled 79 patients undergoing elective cardiac surgery. Simultaneous assessments using ROTEM (EXTEM, INTEM, FIBTEM, PLTEM), PA, and standard coagulation tests were performed. The primary endpoint was the correlation between haemostatic parameters and the Hb/kg Index. Diagnostic accuracy for hypofibrinogenaemia (<2.5 g/L) and thrombocytopenia (<150 × 10^9^/L) was assessed using Receiver Operating Characteristic (ROC) analysis.

**Results:**

Post-CPB platelet count and fibrinogen decreased significantly (p < 0.001). However, a notable dissociation was found: neither platelet count, PLTEM, PA parameters nor standard clotting times correlated with the Hb/kg Index (p > 0.05). In contrast, viscoelastic measures of clot firmness (FIBTEM A10) and fibrinogen levels significantly predicted blood loss. FIBTEM A10 (12 mm) demonstrated excellent accuracy for hypofibrinogenaemia (AUC = 0.888), providing a sensitivity of 96% and a negative predictive value of 97.4%.

**Conclusion:**

Post-CPB bleeding is primarily driven by reduced clot firmness and fibrinogen deficiency rather than by platelet count or aggregation defects. FIBTEM A10 is a superior rapid detector of hypofibrinogenaemia. Transfusion algorithms should prioritize the maintenance of functional clot firmness over the correction of static platelet numbers.

## Introduction

Although the incidence of major blood loss after cardiac surgery has decreased in recent decades, postoperative bleeding after cardiopulmonary bypass (CPB) remains common complication.^1,2^ This adverse event prolongs intensive care unit (ICU) stays, and significantly increases morbidity and mortality.^3,4^ The etiology of bleeding is multifactorial: approximately two-thirds of cases are related to surgical sources, while the remaining third is attributed to coagulopathy.^5^ The latter typically results from haemodilution due to bypass circuit priming, consumption of coagulation factors, tissue factor activation of the extrinsic system, and contact activation of the intrinsic system on circuit components.^6,7^ Additionally, residual unfractionated heparin contributes to haemostatic disorders. Heparin binds not only to antithrombin but also to plasma proteins and endothelial cells. Since protamine neutralises only the circulating fraction, the subsequent release of this sequestered heparin, known as “heparin rebound”, can cause recurrent hypocoagulability.^8,9^ Consequently, these combined factors lead to profound haemostatic disturbances, evidenced by prolonged prothrombin time (PT) and activated partial thromboplastin time (aPTT), as well as significant decreases in fibrinogen concentration, platelet count, and platelet function.^10^ The standard laboratory tests (SLT) used to manage perioperative bleeding include PT, aPTT, fibrinogen level (Clauss method), and platelet count. Except for platelet count, these tests require plasma separation, resulting in a turnaround time of 30 to 90 min.^11^ However, the limitations of SLTs extend beyond latency. While the Clauss method provides a quantitative assessment of fibrinogen, it does not capture the functional adequacy of clot formation.^12^ Similarly, a standard platelet count offers only a quantitative measure and fails to detect qualitative dysfunction induced by antiplatelet therapy or extracorporeal circulation.^13,14^ While light transmission aggregometry (LTA) remains the gold standard for assessing platelet reactivity, its use is limited by technical complexity and prolonged turnaround time.^15^ In contrast, rotational thromboelastometry (ROTEM) evaluates whole blood clot dynamics within 10–15 min, enabling rapid identification of fibrinogen deficiency and platelet dysfunction.^4,16–19^ This rapid turnaround allows the prompt identification of specific coagulopathies, including fibrinogen deficiency and platelet dysfunction.^20,21^ The ROTEM system utilizes EXTEM (extrinsic), INTEM (intrinsic), and FIBTEM (fibrin-specific) tests to assess coagulation,^20^ while HEPTEM and APTEM tests differentiate heparin-induced coagulopathy and detect rapid hyperfibrinolysis, respectively.^22^ The calculated parameter PLTEM (defined as EXTEM minus FIBTEM) is widely used to isolate the platelet contribution to clot firmness.^23^ Accurate estimation of blood loss is essential for validating these hemostatic assays. However, traditional methods based on chest tube drainage volume are often confounded by haemodilution and fail to account for patient weight. To ensure a precise and objective assessment, the Hb/kg Index was used as the primary endpoint, as it reflects the actual hemoglobin mass loss normalized to weight.^24^

In this study, we aimed to evaluate the diagnostic accuracy of viscoelastic tests (ROTEM) compared to standard laboratory parameters and platelet aggregometry (PA) in predicting perioperative blood loss in patients undergoing elective cardiac surgery. Specifically, we sought to determine whether functional clot firmness parameters or platelet counts correlate better with blood loss defined by the Hb/kg Index, and to define optimal ROTEM cut-off values for the rapid detection of post-CPB hypofibrinogenaemia and thrombocytopenia.

### Materials and methods

This prospective observational study was conducted in the Department of Cardiac Surgery and the Department of Anaesthesiology (Intensive Care Unit) at the Hospital of the Lithuanian University of Health Sciences, Kaunas, Lithuania, from October 2023 to November 2024. From October 2023 to November 2024, 79 adult patients undergoing elective cardiac surgery with CPB we enrolled. Exclusion criteria were emergency procedures, reoperations, aortic dissection, heart transplantation, known haematological disorders, and severe hepatic or renal failure.

### Ethical approval

The study was approved by the Kaunas Regional Biomedical Research Committee (Approval No. BE-2-53, August 2023). Written informed consent was obtained from all participants prior to inclusion.

### Outcomes and definitions

Patients discontinued antiplatelet therapy at least 5–7 days before surgery. General anaesthesia and CPB followed standard institutional protocols. The primary endpoint was perioperative haemoglobin mass loss normalised to body weight (Hb/kg Index), calculated for the period up to 18 h postoperatively according to the methodology previously validated by our group.^24^ Hypofibrinogenaemia was defined as a plasma fibrinogen level below 2.5 g/L. This threshold was selected because preoperative levels below this limit are significantly associated with increased postoperative bleeding.^25^ Furthermore, this level is established as a clinically relevant intervention point: Ranucci et al identified it as a pragmatic trigger for early deficit detection,^26^ while in another study it served as the therapeutic target in high-profile randomized trials for actively bleeding patients.^27^ Thrombocytopenia was defined as a platelet count below 150 × 10^9^/L.^28^ This threshold identifies patients with mild thrombocytopenia, a range that Griffin et al demonstrated is part of a continuum of risk, where decreasing platelet counts are independently associated with increased mortality, infection, and acute kidney injury.^29^

### Data collection

Simultaneous blood sampling for ROTEM, PA, and standard laboratory tests was performed at two specific time points: at baseline (before skin incision) and after surgery (following heparin reversal with protamine).

### Viscoelastic testing

Viscoelastic haemostatic assays were performed using the fully automated rotational thromboelastometry system (ROTEM® *sigma*, TEM International GmbH, Munich, Germany) with single-use cartridges. The device was located in the intensive care unit (ICU), providing for point-of-care (POC) analysis by trained medical staff. Testing was conducted at 37 °C using citrated whole blood samples collected at baseline and after heparin reversal. HEPTEM was performed only once, after surgery. The following assays were performed:

- EXTEM: assessment of the extrinsic coagulation pathway initiated by tissue factor.
- INTEM: assessment of the intrinsic coagulation pathway initiated by contact activation.
- FIBTEM: assessment of the fibrinogen contribution to clot formation by activating the extrinsic pathway with platelet inhibition (cytochalasin D).
- HEPTEM: assessment of the intrinsic pathway in the presence of heparinase to neutralize unfractionated heparin. The INTEM/HEPTEM comparison was used to detect residual heparin. The primary variables analyzed were clot amplitude at 10 min (A10) and Maximum Clot Firmness (MCF). Additionally, the specific platelet contribution to clot firmness (PLTEM) was calculated as the arithmetic difference between EXTEM and FIBTEM (PLTEM = EXTEM – FIBTEM).^23^

### Platelet aggregation testing

Platelet function was evaluated using light transmission aggregometry (LTA) on an 8-channel TA-8 V Thrombo-Aggregometer (SD Medical, France). Venous blood samples were collected into 3.2% sodium citrate tubes (BD Vacutainer, Franklin Lakes, NJ, USA) and transported to the Molecular Cardiology Laboratory at the Institute of Cardiology, Lithuanian University of Health Sciences at room temperature within 1 h of venipuncture. Platelet-rich plasma (PRP) was prepared by centrifuging whole blood at 100 × *g* for 10 min, while platelet-poor plasma (PPP) was prepared by centrifuging at 2500 × g for 10 min. PPP was used as the blank control to standardize 100% light transmission. Aggregation was induced by incubating 450 μl of PRP with 10 μl of agonist. The agonists used were: (1) adenosine diphosphate (ADP, final concentration 5 μl; Chrono-Log, Havertown, Pennsylvania, USA), (2) epinephrine (final concentration 10 μl). Traces were recorded in real-time using the device software.

### Statistical analysis

Continuous variables were assessed for normality using the Shapiro–Wilk test. Normally distributed data are presented as mean ± standard deviation (SD), while non-normally distributed data are presented as median and interquartile range (IQR). Categorical variables are reported as frequencies and percentages. Comparisons between preoperative and postoperative values were performed using the Wilcoxon signed-rank test. Correlations between ROTEM parameters, SLT, and PA were evaluated using Spearman's rank correlation coefficients. Receiver operating characteristic (ROC) curve analysis was performed to evaluate the diagnostic accuracy of ROTEM parameters for detecting thrombocytopenia and hypofibrinogenaemia. Areas under the curve (AUCs) with 95% confidence intervals (CIs) were calculated. Optimal cut-off values were determined using Youden's index. Sensitivity, specificity, positive predictive value (PPV), and negative predictive value (NPV) were calculated based on the observed prevalence in the study cohort. All statistical analyses were performed using IBM SPSS Statistics, version 30.0 (IBM Corp., Armonk, NY, USA). A p-value < 0.05 was considered statistically significant.

## Results

The demographic and clinical data are shown in Table 1. The median age was 67 years (IQR 62-72) and the mean BMI was 29.1 ± 5.1 kg/m^2^. The median EuroSCORE II was 2.3 (IQR 1.3-4.0). Surgical procedures included coronary artery bypass grafting (CABG) in 38 patients (48.1%), valve repair or replacement in 26 (32.9%), and combined surgery in 15 (19.0%). The median CPB time was 88 min (IQR 76-121.5). The primary endpoint, perioperative blood loss measured by the Hb/kg index was 1.92 ± 0.72 Hb/kg. Hemostatic profile before and after surgery are summarized in Supplementary Table S1. Standard laboratory tests demonstrated significant reduction after surgery: platelet count decreased by 26% (median 214 to 158 × 10^9^/L, *p* < 0.001) and fibrinogen levels dropped by 26% (median 3.65 to 2.71 g/L, *p* < 0.001). Postoperatively, the prevalence of thrombocytopenia (< 150 × 10^9^/L) was 35.4% (28/79), and hypofibrinogenaemia (< 2.5 g/L) was observed in 30.4% (24/79) of patients. INR increased from 1.00 to 1.20 (*p* < 0.001) and aPTT was prolonged from 33.7 to 42.8 s (*p* < 0.001). Despite significant reductions in platelet count and fibrinogen, viscoelastic parameters were better preserved. Although median EXTEM A10 decreased (59 vs 57 mm, *p* < 0.001), values remained largely within the normal range. Across all ROTEM parameters, early clot amplitudes (A5 and A10) showed excellent linear correlations with Maximum Clot Firmness (MCF) (r > 0.95, p < 0.001). Given this strong association and the clinical advantage of obtaining results much earlier, the following analysis focuses primarily on A10 parameters. HEPTEM was performed only once after surgery. The median HEPTEM clotting time (CT) was 185 s (IQR 175-191). Comparison of HEPTEM with the paired INTEM CT (median 195 s, IQR 187-212) revealed a statistically significant difference (*p* < 0.001). While the median reduction was only 10 s, analysis of individual variation showed that 26 patients (33%) had an INTEM/HEPTEM CT ratio greater than 1.1, suggesting a mild residual heparin effect. Unlike other clot firmness parameters, EXTEM CT showed no significant correlation with INR (ρ = -0.183, p = 0.11). Conversely, a moderate positive correlation was observed between INTEM CT and aPTT after surgery (ρ = 0.331, p = 0.003).

**Table 1.** Baseline Characteristics and Procedural Data (N = 79).

Age (years)	67 (62-72)
Sex, male	58 (73.4%)
Weight (kg)	86.0 ± 16.0
Body mass index (kg/m^2^)	29.1 ± 5.1
EuroSCORE II	2.3 (1.3-4.0)
**Type of surgery:**	
CABG	38 (48.1%)
Valve	26 (32.9%)
Combined	15 (19%)
CPB time, min	88 (76-121.5)
Cross-clamp time, min	51 (39-71.5)
**Primary endpoint** (Blood loss)	
Hb/kg	1.92 ± 0.72

Data are presented as mean ± standard deviation (SD) for normally distributed variables, median (interquartile range, IQR) for non-normally distributed variables, and n (%) for categorical variables. CABG: Coronary Artery Bypass Grafting; CPB: Cardiopulmonary Bypass; Hb/kg: Hemoglobin mass loss per kilogram of body weight;

**Inter-assay correlations for postoperative parameters** are presented in the Supplementary Table S2. ROTEM parameters demonstrated expected associations with PA and standard laboratory tests. Preoperatively, EXTEM A10 and INTEM A10 were significantly correlated with platelet count (ρ = 0.387 and ρ = 0.387, *p* < 0.001). These associations became stronger postoperatively, with platelet count showing strong correlations with EXTEM A10 (ρ = 0.581) as well as INTEM A10 (ρ = 0.486) (all *p* < 0.001). PLTEM demonstrated statistically significant positive correlations with platelet count at both time points (preoperative A10: ρ = 0.410; postoperative A10: ρ = 0.512; all *p* < 0.01). Platelet aggregation (PA) parameters demonstrated no significant correlation with platelet count at any time point. Neither ADP-induced (ρ = 0.028, *p* = 0.804) nor epinephrine-induced aggregation (ρ = -0.046, *p* = 0.685) correlated with platelet count before surgery, and this lack of association persisted after surgery. FIBTEM A10 demonstrated a robust linear relationship with plasma fibrinogen concentration, particularly after surgery (*r* = 0.764, *p* < 0.01).

### Correlation with Hb/kg index

**Figure 1.**
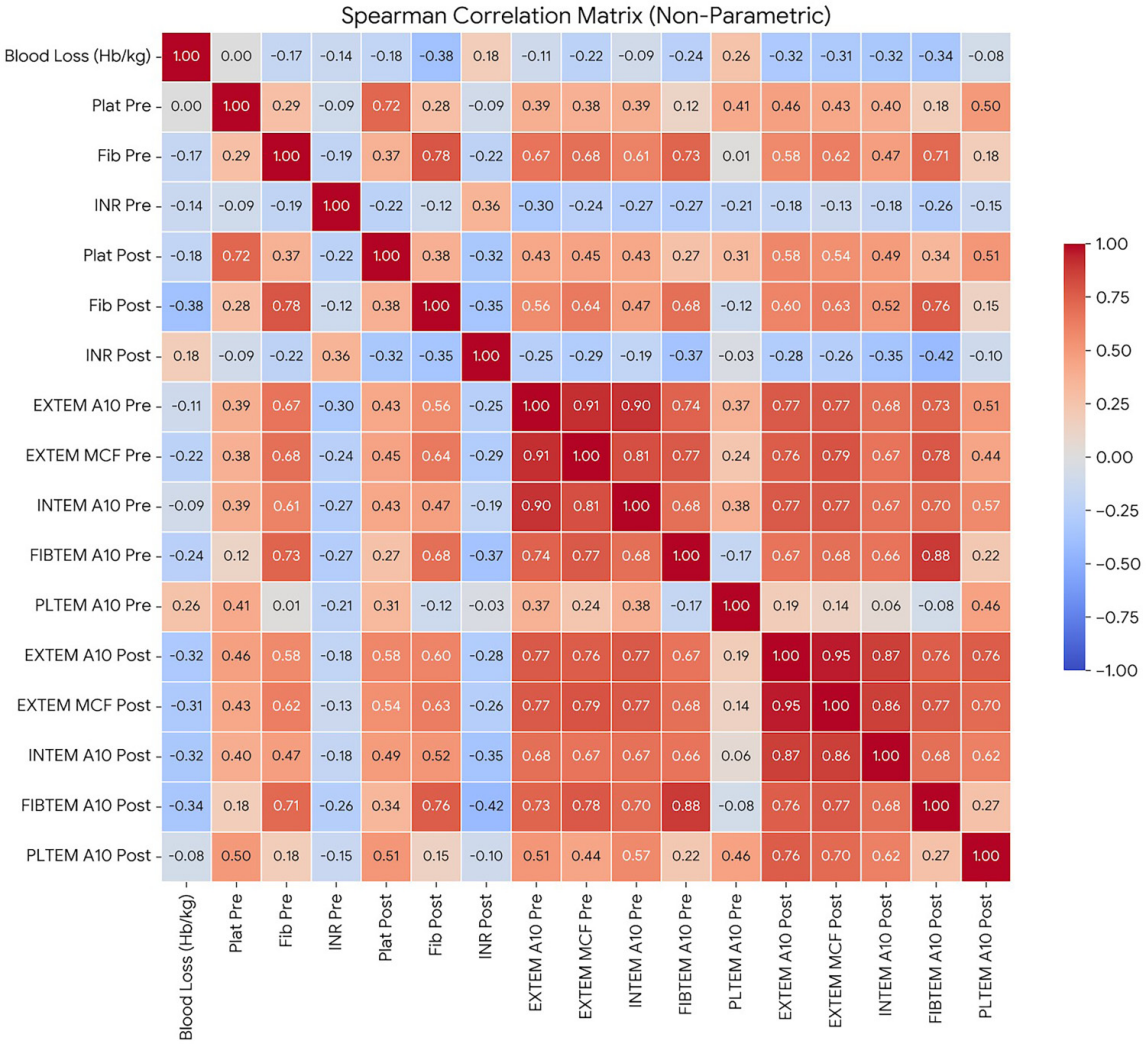
Correlation coefficients for perioperative parameters with the Hb/kg index.

The detailed correlation coefficients for all perioperative parameters with the Hb/kg index are provided in Table 2 and visually summarized in Figure 1. In contrast to platelets, postoperative fibrinogen levels demonstrated a significant inverse correlation with the Hb/kg index (ρ = -0.379, *p* < 0.001). Similarly, preoperative FIBTEM A10 showed significant inverse correlation with the Hb/kg index (ρ = -0.242, *p* = 0.036), which became stronger after surgery (ρ = -0.338, *p* = 0.002). Significant inverse correlations were also found between the Hb/kg index and postoperative EXTEM A10 and INTEM A10 (ρ = -0.318 and ρ = -0.322, both *p* < 0.01). In contrast, preoperative EXTEM and INTEM parameters were not associated with Hb/kg loss (all *p* > 0.05). HEPTEM clot firmness demonstrated a significant inverse correlation with Hb/kg loss (A10: ρ = -0.270, *p* = 0.016; MCF: ρ = -0.298, *p* = 0.008) similar to that observed with INTEM. Although a weak positive correlation was observed between preoperative PLTEM A10 and the Hb/kg index (ρ = 0.263, *p* = 0.019), no significant association was found after surgery. For blood loss prediction, preoperative ADP-induced PA showed only a non-significant trend with the Hb/kg index (ρ = -0.190, *p* = 0.094). Postoperatively, neither PA parameter demonstrated a significant association with the Hb/kg index (ADP-induced: ρ = -0.104, *p* = 0.359; epinephrine-induced: ρ = -0.083, *p* = 0.469). Moreover, the INR, aPTT, and postoperative platelet count demonstrated no significant correlation with the Hb/kg index (*p* > 0.05).

**Table 2.** Correlations of perioperative hemostatic profile with the Hb/kg Index loss.

Parameter	Preoperative		Postoperative	
	rho	*p-value*	rho	*p-value*
**Standard laboratory tests**				
Platelets (×10^9^/L)	0.002	0.987	−0.184	0.105
Fibrinogen (g/L)	−0.168	0.139	−0.379	< 0.001
INR	−0.138	0.227	0.183	0.107
aPTT	0.049	0.669	0.030	0.792
**ROTEM parameters**				
EXTEM CT	0.046	0.685	0.105	0.355
EXTEM A10	−0.107	0.349	−0.318	0.004
EXTEM MCF	−0.220	0.052	−0.311	0.005
INTEM CT	−0.153	0.180	0.196	0.084
INTEM A10	−0.091	0.424	−0.322	0.004
INTEM MCF	−0.182	0.109	−0.343	0.002
FIBTEM A10	−0.242	0.032	−0.338	0.002
FIBTEM MCF	−0.236	0.036	−0.315	0.005
PLTEM A10	0.263	0.019	−0.083	0.465
PLTEM MCF	0.167	0.142	−0.077	0.499
**Platelet aggregation**				
ADP-induced PA	−0.190	0.094	−0.104	0.359
Epinephrine-induced PA	−0.023	0.840	−0.083	0.469

Abbreviations: A10: clot amplitude at 10 min; ADP: Adenosine diphosphate; aPTT: activated Partial Thromboplastin Time; INR: International Normalized Ratio; MCF: Maximum Clot Firmness; PA: Platelet Aggregation; PLTEM: calculated platelet contribution to clot firmness (EXTEM – FIBTEM).

**Figure 2.**
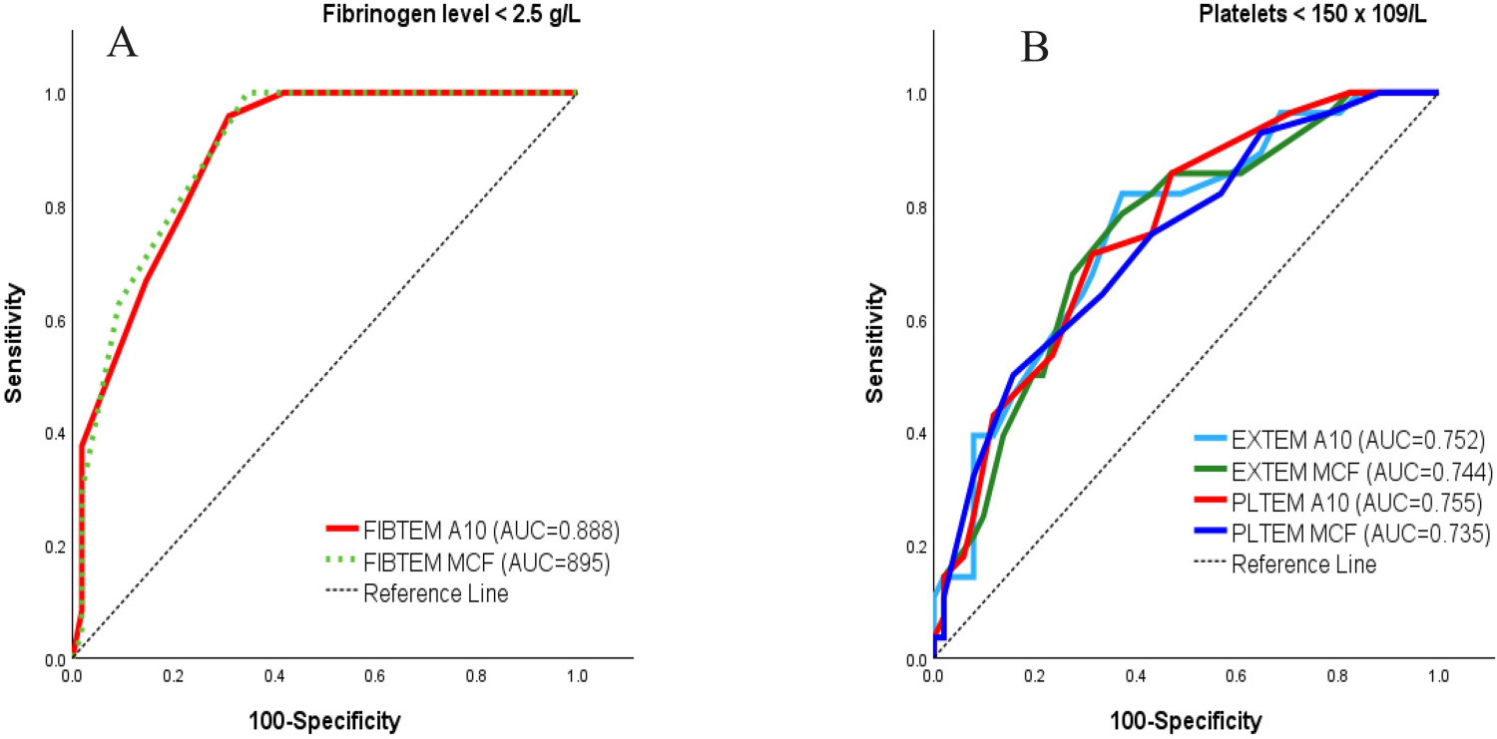
ROC-curves for ROTEM parameters.

**The diagnostic accuracy of ROTEM** in detecting specific coagulopathies defined by clinical thresholds (hypofibrinogenaemia < 2.5 g/L and thrombocytopenia < 150 × 10^9^/L) was evaluated (Table 3). *Prediction of hypofibrinogenaemia.* FIBTEM A10 demonstrated excellent diagnostic accuracy for detecting hypofibrinogenaemia (< 2.5 g/L) with an AUC of 0.888 (95% CI 0.797-0.979, *p* < 0.001) (Figure 2A). ROC analysis identified an optimal cut-off value of 12 mm (maximum Youden's index = 0.649), providing high sensitivity (96%) and specificity (69.1%). At this threshold, the negative predictive value (NPV) was 97.4%, indicating that FIBTEM A10 > 12 mm effectively rules out clinically relevant hypofibrinogenaemia in this cohort.

**Table 3.** Diagnostic Accuracy and Cut-off Values of Postoperative ROTEM Parameters for Predicting Hypofibrinogenaemia and Thrombocytopenia.

	ROTEM Parameters	Cut-Off (mm)	Sensitivity	Specificity	PPV	NPV	AUC	95% CI	p-Value
**Fibrinogen** (2.5 g/l)	FIBTEM A10	≤ 12	95.8	69.1	57.5	97.4	0.888	0.797-0.979	< 0.001
	FIBTEM MCF	≤ 14	100.0	65.5	55.8	100.0	0.895	0.807-0.984	< 0.001
**Platelets** (< 150 * 10^9^/L)	EXTEM A10	≤ 57	82.1	62.7	54.8	86.5	0.752	0.634-0.870	< 0.001
	EXTEM MCF	≤ 64	78.6	62.7	53.7	84.2	0.744	0.625-0.872	< 0.001
	PLTEM A10	≤ 43	71.4	68.6	55.6	81.4	0.755	0.638-0.872	< 0.001
	PLTEM MCF	≤ 48	50.0	84.3	63.6	75.4	0.735	0.614-0.856	< 0.001

Abbreviations: A10: clot amplitude at 10 min; AUC: area under the curve; CI: confidence interval; MCF: maximum clot firmness; NPV: negative predictive value; PLTEM: calculated platelet contribution to clot firmness (EXTEM – FIBTEM); PPV: positive predictive value;

*Prediction of thrombocytopenia.* For predicting thrombocytopenia after surgery (< 150 × 10^9^/L), viscoelastic parameters showed moderate diagnostic performance (Figure 2B). PLTEM A10 (AUC = 0.755) and EXTEM A10 (AUC = 0.752) were the most informative predictors, while INTEM A10 demonstrated lower discrimination (AUC = 0.670). Using an EXTEM A10 cut-off of 57 mm (positive if ≤ 57 mm) yielded a sensitivity of 82% and specificity of 63% (PPV = 55%, NPV = 87%). A PLTEM A10 cut-off of 43 mm provided a more balanced profile, with sensitivity of 71% and specificity of 69% (PPV = 56%, NPV = 81%).

## Discussion

The principal finding of this study demonstrates that early post-CPB coagulopathy and subsequent blood loss are driven primarily by fibrinogen deficiency rather than platelet dysfunction. We found that viscoelastic parameters of clot firmness (FIBTEM A10) strongly correlated with the Hb/kg Index and showed excellent diagnostic accuracy for hypofibrinogenaemia. In contrast, despite observing significant thrombocytopenia and reduced PA were observed after surgery, neither standard PA nor the calculated ROTEM platelet component (PLTEM) showed a significant association with blood loss in this cohort. Furthermore, we validated the Hb/kg Index as a more physiological metric for quantifying blood loss compared to traditional volume-based measurements.^24^

### The Role of Fibrinogen and Viscoelastic Monitoring

Our results support the concept that fibrinogen is the “first factor to fall” in cardiac surgery involving CPB.^30^ We observed a 26% reduction in plasma fibrinogen levels, with nearly one-third of patients developing clinically significant hypofibrinogenaemia. This reduction is related to the combined effects of haemodilution,^31,32^ consumptive coagulopathy induced by contact activation,^33^ and hyperfibrinolysis caused by systemic inflammation.^34^ The strong correlation between FIBTEM A10 and both the Clauss fibrinogen level (r = 0.783) and the Hb/kg Index (ρ = -0.338) supports the use of ROTEM as a first-line guide for haemostatic resuscitation. ROC analysis identified a FIBTEM A10 cut-off of 12 mm as a highly sensitive predictor of hypofibrinogenaemia (AUC 0.888; 95% CI 0.797-0.979). This threshold aligns with Waldén et al,^25^ who identified fibrinogen as an independent predictor of bleeding, and with Ranucci et al, who established similar trigger values.^26^ The previous 2017 EACTS/EACTA Guidelines on patient blood management for adult cardiac surgery suggested a conservative fibrinogen trigger of <1.5 g/L.^30^ In contrast, the 2024 EACTS/EACTAIC Guidelines on patient blood management (in collaboration with EBCP) validate the need for higher thresholds, advocating a target of ≥2.0 g/L and issuing a Class IIa recommendation for viscoelastic-guided therapy.^35^ Our data provide rigorous validation for this updated strategy. Using the weight-adjusted blood loss calculation (Hb/kg Index) as a more precise physiological metric, we demonstrated that bleeding risk increases significantly at <2.5 g/L (corresponding to FIBTEM A10 of 12 mm). Furthermore, the high negative predictive value (97.4%) observed in our study indicates that a normal FIBTEM trace effectively rules out fibrinogen deficiency, enabling clinicians to consider other potential causes of bleeding.

### Platelet Count versus Function

A notable finding of our study is the dissociation between platelet function and blood loss. Despite a significant postoperative decrease in platelet count and a marked inhibition of aggregation (ADP-induced PA decreased by 23%; epinephrine-induced PA by 57%), aggregometry failed to predict blood loss. Similarly, PLTEM, which reflects the platelet contribution to clot firmness, correlated with platelet count but not with the Hb/kg Index. This dynamic, where PLTEM remains stable despite a significant drop in platelet count, mirrors findings by Baryshnikova et al, identified platelet dysfunction as a primary predictor of bleeding, we found no association when using the Hb/kg index as the outcome measure.^36^ This discrepancy highlights the limitations of assessing platelets in isolation to predict a dynamic physiological process. While Griffin et al describe thrombocytopenia as a continuum of risk,^29^ our results suggest that in the immediate post-protamine period, maintaining a specific clot strength (as measured by FIBTEM) is more relevant for haemostasis than targeting an arbitrary platelet count. As long as the fibrin mesh is compromised (low FIBTEM), the impact of platelet function may be diminished. This supports the “fibrinogen-dependent” nature of clot firmness observed in randomised trials, where fibrinogen supplementation effectively reduced bleeding even in the presence of platelet dysfunction.^27^ Thus, clinicians should avoid treatment decisions based solely on isolated aggregation values or platelet counts; instead, the focus should remain on clinical bleeding and restoring functional clot firmness (FIBTEM).

### The Hb/kg Index

Accurate quantification of perioperative blood loss remains a significant challenge. Although numerous calculation methods have been described,^37–40^ none is generally accepted as a “gold standard”.^37^ Conventional estimation methods based on volumetric or visual assessments of chest tube output are inaccurate and subjective,^41^ because CTD confounded by the variable content of serous fluid, residual irrigation, and haemodilution.^42^ Moreover, absolute volume-based measurements often overlook a patient's weight. The clinical impact of blood loss is relative to estimated blood volume; for example, the same 1000 mL loss has markedly different haemodynamic consequences for a patient weighing 80 kg compared to one weighing 120 kg. Therefore, a normalised, physiological parameter is required to evaluate bleeding severity accurately. To address these limitations, we used the Hb/kg Index to quantify blood loss. By normalising haemoglobin mass loss to body weight, as validated in our recent work,^24^ we minimised the confounding effects of haemodilution and accounted for individual constitution. In this study the Hb/kg Index demonstrated a statistically significant correlation with clot firmness (FIBTEM A10) and post-surgery fibrinogen level, in contrast to the lack of association with INR, aPTT, PLTEM, platelet counts, or platelet aggregation.

### Limitations

This study has several limitations. First, it was a single-centre observational study with a relatively small sample size, which may limit the power to detect subtle associations, particularly for platelet aggregation. Second, while we detected a mild residual heparin effect in 33% of patients via HEPTEM, its clinical contribution appeared minor compared to the fibrinogen deficit.

## Conclusion

In patients undergoing elective cardiac surgery, post-CPB bleeding is significantly associated with reduced clot firmness and hypofibrinogenaemia, but not with platelet aggregation defects measured by PA. FIBTEM A10 provides a rapid and accurate tool for detecting relevant hypofibrinogenaemia. Consequently, haemostatic algorithms in this setting should prioritize the restoration of fibrinogen levels and clot firmness, while the routine use of platelet function testing for bleeding risk stratification requires further investigation.

## Supplemental Material

sj-docx-1-cat-10.1177_10760296261432450 - Supplemental material for A Prospective Analysis of Viscoelastic Assays, Platelet Aggregometry, and Standard Laboratory Tests in Predicting Perioperative Blood Loss in Cardiac Surgery Supplemental material, sj-docx-1-cat-10.1177_10760296261432450 for A Prospective Analysis of Viscoelastic Assays, Platelet Aggregometry, and Standard Laboratory Tests in Predicting Perioperative Blood Loss in Cardiac Surgery by Yerlan Orazymbetov, Serik Aitaliyev, Povilas Jakuška, Audronė Veikutienė, Tadas Lenkutis, Rassul Zhumagaliyev, Vilius Skipskis, Yerik Aitaliyev, Aušra Saudargienė and Rimantas Benetis in Clinical and Applied Thrombosis/Hemostasis

## References

[1] Vonk ABA Meesters MI Van Dijk WB , et al. Ten-year patterns in blood product utilization during cardiothoracic surgery with cardiopulmonary bypass in a tertiary hospital. Transfusion (Paris). 2014;54(1):2608–2616. doi:10.1111/trf.12522 24372139

[2] Colson PH Gaudard P Fellahi JL , et al. Active bleeding after cardiac surgery: A prospective observational multicenter study. PLoS One. 2016;11(9):1–14. doi:10.1371/journal.pone.0162396 PMC5010224 27588817

[3] Christensen MC Dziewior F Kempel A Von Heymann C . Increased chest tube drainage is independently associated with adverse outcome after cardiac surgery. J Cardiothorac Vasc Anesth. 2012;26(1):46–51. doi:10.1053/j.jvca.2011.09.021 22100857

[4] Görlinger K Shore-Lesserson L Dirkmann D Hanke AA Rahe-Meyer N Tanaka KA . Management of hemorrhage in cardiothoracic surgery. J Cardiothorac Vasc Anesth. 2013;27(4 SUPPL.):S20–S34. doi:10.1053/j.jvca.2013.05.014 23910533

[5] Moulton MJ Creswell LL Mackey ME Cox JL Rosenbloom M . Reexploration for bleeding is a risk factor for adverse outcomes after cardiac operations. J Thorac Cardiovasc Surg. 1996;111(5):1037–1083. doi:10.1016/S0022-5223(96)70380-X 8622301

[6] Erdoes G Faraoni D Koster A Steiner ME Ghadimi K Levy JH . Perioperative considerations in management of the severely bleeding coagulopathic patient. Anesthesiology. Lippincott Williams and Wilkins. 2023;138(5):535–560. doi:10.1097/ALN.0000000000004520 PMC10373857 36862401

[7] Ranucci M Baryshnikova E Ciotti E Ranucci M Silvetti S . Hemodilution on cardiopulmonary bypass: Thromboelastography patterns and coagulation-related outcomes. J Cardiothorac Vasc Anesth. 2017;31(5):1588–1594. doi:10.1053/j.jvca.2017.04.014 28778772

[8] Ichikawa J Kodaka M Nishiyama K Hirasaki Y Ozaki M Komori M . Reappearance of circulating heparin in whole blood heparin concentration-based management does not correlate with postoperative bleeding after cardiac surgery. J Cardiothorac Vasc Anesth. 2014;28(4):1003–1007. doi:10.1053/j.jvca.2013.10.010 24508375

[9] Teoh KHT Young E Blackall MH Roberts RS Hirsh J . Can extra protamine eliminate heparin rebound following cardiopulmonary bypass surgery? J Thoracic and Cardiovascular Surgery. 2004;128(2):211–219. doi:10.1016/j.jtcvs.2003.12.023 15282457

[10] Höfer J Fries D Solomon C Velik-Salchner C Ausserer J . A snapshot of coagulopathy after cardiopulmonary bypass. Clinical and Applied Thrombosis/Hemostasis. 2016;22(6):505–511. doi:10.1177/1076029616651146 27268940

[11] Tanaka KA Bolliger D Vadlamudi R Nimmo A . Rotational thromboelastometry (ROTEM)-based coagulation management in cardiac surgery and major trauma. J Cardiothorac Vasc Anesth. 2012;26(6):1083–1093. doi:10.1053/j.jvca.2012.06.015 22863406

[12] Fenger-Eriksen C Moore GW Rangarajan S Ingerslev J Sørensen B . Fibrinogen estimates are influenced by methods of measurement and hemodilution with colloid plasma expanders. Transfusion (Paris). 2010;50(12):2571–2576. doi:10.1111/j.1537-2995.2010.02752.x 20576008

[13] Solomon C Ranucci M Hochleitner G Schochl H Schlimp CJ . Assessing the methodology for calculating platelet contribution to clot strength (platelet component) in thromboelastometry and thrombelastography. Anesth Analg. Lippincott Williams and Wilkins. 2015;121(4):868–878. doi:10.1213/ANE.0000000000000859 PMC4568902 26378699

[14] Ranucci M Baryshnikova E Castelvecchio S Pelissero G . Major bleeding, transfusions, and anemia: The deadly triad of cardiac surgery. Annals of Thoracic Surgery. 2013;96(2):478–485. doi:10.1016/j.athoracsur.2013.03.015 23673069

[15] Paniccia R Antonucci E Maggini N , et al. Light Transmittance Aggregometry Induced by Different Concentrations of Adenosine Diphosphate to Monitor Clopidogrel Therapy: A Methodological Study. 10.1097/FTD.0b013e3182052ff4 21192314

[16] Görlinger K Pérez-Ferrer A Dirkmann D , et al. The role of evidence-based algorithms for rotational thromboelastometry-guided bleeding management. Korean J Anesthesiol. 2019;72(4):297–322. doi:10.4097/kja.19169 31096732 PMC6676023

[17] Kozek-Langenecker SA Afshari A Albaladejo P , et al. Management of severe perioperative bleeding: Guidelines from the European society of anaesthesiology. Eur J Anaesthesiol. 2013;30(6):270–382. doi:10.1097/EJA.0b013e32835f4d5b 23656742

[18] Schenk B Görlinger K Treml B , et al. A comparison of the new ROTEM ® sigma with its predecessor, the ROTEMdelta. Anaesthesia. 2019;74(3):348–356. doi:10.1111/anae.14542 30575011

[19] Görlinger K Gandhi A . Utility of platelet function testing in cardiac surgery in 2021. Journal of Cardiac Critical Care TSS. 2021;5(02):084–087. doi:10.1055/s-0041-1732839

[20] Haensig M Kempfert J Kempfert PM Girdauskas E Borger MA Lehmann S . Thrombelastometry guided blood-component therapy after cardiac surgery: A randomized study. BMC Anesthesiol. 2019;19(1):1–10. doi:10.1186/s12871-019-0875-7 31694568 PMC6833285

[21] Gronchi F Perret A Ferrari E , et al. Validation of rotational thromboelastometry during cardiopulmonary bypass: A prospective, observational in-vivo study. Eur J Anaesthesiol. 2014;31(2):68–75. doi:10.1097/EJA.0b013e328363171a 23867776

[22] Haas T Fries D Tanaka KA Asmis L Curry NS Schöchl H . Usefulness of standard plasma coagulation tests in the management of perioperative coagulopathic bleeding: Is there any evidence? Br J Anaesth. 2015;114(2):217–224. doi:10.1093/bja/aeu303 25204698

[23] Olde Engberink RHG Kuiper GJAJM Wetzels RJH , et al. Rapid and correct prediction of thrombocytopenia and hypofibrinogenemia with rotational thromboelastometry in cardiac surgery. J Cardiothorac Vasc Anesth. 2014;28(2):210–216. doi:10.1053/j.jvca.2013.12.004 24630470

[24] Orazymbetov Y Aitaliyev S Jakuška P , et al. Calculation of blood loss in cardiac surgery: How should we monitor? Perfusion (United Kingdom). 2025;0(0):1–8. doi:10.1177/02676591251370110 PMC13144624 40832907

[25] Waldén K Jeppsson A Nasic S Backlund E Karlsson M . Low preoperative fibrinogen plasma concentration is associated with excessive bleeding after cardiac operations. Ann Thorac Surg. 2014;97(4):1199–1206. doi:10.1016/j.athoracsur.2013.11.064 24507940

[26] Ranucci M Jeppsson A Baryshnikova E . Pre-operative fibrinogen supplementation in cardiac surgery patients: An evaluation of different trigger values. Acta Anaesthesiol Scand. 2015;59(4):427–433. doi:10.1111/aas.12469 25600583

[27] Bilecen S De Groot JAH Kalkman CJ , et al. Effect of fibrinogen concentrate on intraoperative blood loss among patients with intraoperative bleeding during high-risk cardiac surgery: A randomized clinical trial. JAMA. 2017;317(7):738–747. doi:10.1001/jama.2016.21037 28241354

[28] Izak M Bussel JB . Management of thrombocytopenia. F1000Prime Rep. 2014;6(45):1–10. doi:10.12703/P6-45 24991422 PMC4047949

[29] Griffin BR Bronsert M Reece TB , et al. Thrombocytopenia after cardiopulmonary bypass is associated with increased morbidity and mortality. Ann Thorac Surg. 2020;110(1):50–57. doi:10.1016/j.athoracsur 31816284 PMC7279614

[30] Pagano D Milojevic M Meesters MI , et al. 2017 EACTS/EACTA guidelines on patient blood management for adult cardiac surgery: The task force on patient blood management for adult cardiac surgery of the European association for cardio-thoracic surgery (EACTS) and the European association of cardiothoracic anaesthesiology (EACTA). European J Cardio-Thoracic Surgery. 2018;53(1):79–111. doi:10.1093/ejcts/ezx325 29029100

[31] Sniecinski RM Chandler WL . Activation of the hemostatic system during cardiopulmonary bypass. Anesth Analg. 2011;113(6):1319–1333. doi:10.1213/ANE.0b013e3182354b7e 22003219

[32] Warner DS Bolliger D Gö K Tanaka KA . Pathophysiology and Treatment of Coagulopathy in Massive Hemorrhage and Hemodilution. Vol 113. 2010. 10.1097/ALN.0b013e3181f22b5a 20881594

[33] Paparella D Brister SJ Buchanan MR . Coagulation disorders of cardiopulmonary bypass: A review. Intensive Care Med. 2004;30(10):1873–1881. doi:10.1007/s00134-004-2388-0 15278267

[34] Mojcik CF Levy JH . Aprotinin and the systemic inflammatory response after cardiopulmonary bypass blood contact with the gaseous interface and bioincom-patible surfaces of the extracorporeal system during CPB. Ann Thorac Surg. 2001;71(2):745–754. 11235755 10.1016/s0003-4975(00)02218-9

[35] Casselman FPA Lance MD Ahmed A , et al. 2024 EACTS/EACTAIC Guidelines on patient blood management in adult cardiac surgery in collaboration with EBCP. Eur J Cardiothorac Surg. 2025;67(5):1–55. doi:10.1093/ejcts/ezae352 PMC12257489 39385500

[36] Baryshnikova E Di Dedda U Ranucci M . Are viscoelastic tests clinically useful to identify platelet-dependent bleeding in high-risk cardiac surgery patients? Anesth Analg. 2022;135(6):1198–1206. doi:10.1213/ANE.0000000000006231 36227767

[37] Jaramillo S Montane-Muntane M Gambus PL Capitan D Navarro-Ripoll R Blasi A . Perioperative blood loss: Estimation of blood volume loss or haemoglobin mass loss? Blood Transfusion. 2020;18(1):20–29. doi:10.2450/2019.0204-19 31855150 PMC7053522

[38] Lopez-Picado A Albinarrate A Barrachina B . Determination of perioperative blood loss: Accuracy or approximation? Anesth Analg. 2017;125(1):280–286. doi:10.1213/ANE.0000000000001992 28368940

[39] Meunier A Petersson A Good L Berlin G . Validation of a haemoglobin dilution method for estimation of blood loss. Vox Sang. 2008;95(2):120–124. doi:10.1111/j.1423-0410.2008.01071.x 18510580

[40] Dyke C Aronson S Dietrich W , et al. Universal definition of perioperative bleeding in adult cardiac surgery. J Thoracic and Cardiovascular Surgery. 2014;147(5):1458–1464. doi:10.1016/j.jtcvs.2013.10.070 24332097

[41] Stoker AD Binder WJ Frasco PE , et al. Estimating surgical blood loss: A review of current strategies in various clinical settings. SAGE Open Med. 2024;12(12):1–6. doi:10.1177/20503121241308302 PMC11650593 39691865

[42] Karimov JH Gillinov AM Schenck L , et al. Incidence of chest tube clogging after cardiac surgery: A single-centre prospective observational study. Eur J Cardiothorac Surg. 2013;44(6):1029–1036. doi:10.1093/ejcts/ezt140 23520232

