# Supplementary material for: A Prospective Analysis of Viscoelastic Assays, Platelet Aggregometry, and Standard Laboratory Tests in Predicting Perioperative Blood Loss in Cardiac Surgery
Source: Clin Appl Thromb Hemost. 2026 Mar 5;32:10760296261432450. doi: 10.1177/10760296261432450 (PMC12966529; doi:10.1177/10760296261432450)
Supplement: sj-docx-1-cat-10.1177_10760296261432450 - Supplemental material for A Prospective Analysis of Viscoelastic Assays, Platelet Aggregometry, and Standard Laboratory Tests in Predicting Perioperative Blood Loss in Cardiac Surgery [file sj-docx-1-cat-10.1177_10760296261432450.docx]

**Supplementary Table S1. Hemostatic Profile before and after surgery (N = 79).**

| **Parameters** | **Baseline** | **After surgery** | **p-value** |
| --- | --- | --- | --- |
| **ROTEM** | | | |
| EXTEM CT, s | 64 (60 – 69) | 63 (60–68) | 0.542 |
| EXTEM A10, mm | 59 (56–62) | 57 (53–61) | < 0.001 |
| EXTEM MCF, mm | 66 (64–69) | 64 (61–68) | < 0.001 |
| INTEM CT, s | 193 (184 – 202) | 195 (186–212) | 0.011 |
| INTEM A10, mm | 55 (53–58) | 53 (50–57) | < 0.001 |
| INTEM MCF, mm | 63 (60–66) | 61 (57–65) | < 0.001 |
| FIBTEM A10, mm | 15 (12–17) | 12 (10–15) | < 0.001 |
| FIBTEM MCF, mm | 16 (14–19) | 14 (12–17) | < 0.001 |
| PLTEM A10, mm | 44 (42–46) | 44 (42–47) | 0.630 |
| PLTEM MCF, mm | 50 (49–53) | 50 (48–52) | 0.195 |
| **Conventional laboratory tests** | | | |
| Platelets (×10^9^/L) | 214 (182–242) | 158 (142–194) | < 0.001 |
| Fibrinogen (g/L) | 3.65 (3.29–4.14) | 2.71 (2.38–3.21) | < 0.001 |
| INR | 1.00 (0.97–1.09) | 1.20 (1.10–1.20) | < 0.001 |
| aPTT | 33.7 (30.6 – 38.0) | 42.8 (37.8 – 47.2) | < 0.001 |
| **Platelet aggregation** | | | |
| ADP-induced PA, % | 73 (64–81) | 56 (44–64) | < 0.001 |
| Epinephrine-induced PA, % | 80 (75–84) | 34 (16–68) | < 0.001 |

P values were calculated using the Wilcoxon signed-rank test. Data are presented as median and interquartile range (IQR). ADP: adenosine diphosphate; aPTT: activated partial thromboplastin time; A10: clot amplitude at 10 min; CT: clotting time; INR: international normalised ratio; MCF: maximum clot firmness; PA: platelet aggregation; PLTEM: calculated platelet contribution to clot firmness.

**Supplementary Table S2. Spearman correlations between postoperative viscoelastic/aggregation parameters and standard laboratory tests**

|  | rho (p-value) | rho (p-value) | rho (p-value) | rho (p-value) |
| --- | --- | --- | --- | --- |
|  | **Platelet count** | **Fibrinogen level (Clauss)** | **INR** | **aPTT** |
| **ROTEM Parameters** | | | | |
| EXTEM CT | 0.265 (0.018) | 0.133 (0.243) | -0.183 (0.107) | -0.001 (0.996) |
| EXTEM A10 | 0.581 (< 0.001) | 0.467 (< 0.001) | -0.278 (0.013) | -0.169 (0.136) |
| EXTEM MCF | 0.542 (< 0.001) | 0.505 (< 0.001) | -0.286 (0.011) | -0.198 (0.081) |
| INTEM CT | -0.097 (0.395) | -0.032 (0.779) | 0.265 (0.018) | 0.331 (0.003) |
| INTEM A10 | 0.486 (< 0.001) | 0.490 (< 0.001) | -0.267 (0.018) | -0.167 (0.141) |
| INTEM MCF | 0.444 (< 0.001) | 0.518 (< 0.001) | -0.273 (0.015) | -0.201 (0.076) |
| FIBTEM A10 | 0.205 (0.070) | 0.764 (< 0.001) | -0.301 (0.007) | -0.237 (0.035) |
| FIBTEM MCF | 0.199 (0.079) | 0.777 (< 0.001) | -0.293 (0.009) | -0.245 (0.029) |
| PLTEM A10 | 0.512 (< 0.001) | -0.019 (0.869) | -0.103 (0.369) | 0.005 (0.963) |
| PLTEM MCF | 0.435 (< 0.001) | 0.061 (0.596) | -0.145 (0.202) | -0.067 (0.559) |
| **Platelet aggregation** | | | | |
| ADP-induced PA | 0.130 (0.254) | 0.046 (0.690) | -0.130 (0.256) | -0.065 (0.570) |
| Epinephrine-induced PA | 0.166 (0.144) | 0.032 (0.779) | 0.028 (0.806) | 0.071 (0.536) |

Abbreviations: A10: clot amplitude at 10 min; ADP: Adenosine diphosphate; aPTT: activated Partial Thromboplastin Time; CT: clotting time; INR: International Normalized Ratio; MCF: Maximum Clot Firmness; PA: Platelet Aggregation; PLTEM: calculated platelet contribution to clot firmness (EXTEM – FIBTEM).
